# The segment-to-frame association in word reading: early effects of the interaction between segmental and suprasegmental information

**DOI:** 10.3389/fpsyg.2015.01612

**Published:** 2015-10-20

**Authors:** Simone Sulpizio, Remo Job

**Affiliations:** ^1^Department of Psychology and Cognitive Science, University of TrentoTrento, Italy; ^2^Fondazione Marica De Vincenzi ONLUSTrento, Italy

**Keywords:** stress assignment, phonological encoding, masked priming, reading aloud, articulation

## Abstract

In four reading aloud experiments we investigated the operations occurring at the level of the phonological buffer by manipulating stress and phoneme information. In all experiments we adopted a masked priming paradigm with three-syllable Italian word targets. Experiments 1 and 2 tested the effect of pure segmental (e.g., fe%%%% – FEcola) and pure suprasegmental (CInema – FEcola) overlap, respectively. Experiments 3 and 4 tested the joint manipulation of segmental and suprasegmental information, by using prime-target pairs that shared the first syllable and did or did not share their stress pattern (e.g., FEgato – FEcola vs. feNIce – FEcola). The results showed that both segmental and suprasegmental primes affect reading at an abstract phonological level. Moreover, the joint manipulation of stress and phonemes showed an asymmetric pattern for different stress patterns, suggesting that the phonemic and the stress systems address the articulation planning through a process that starts as soon as the relevant information about the to-be-planned unit is active.

## Introduction

Reading aloud involves computing the sound of a word from its visually presented form. In order to carry out such process the execution of multiple operations is required, e.g., perceiving the written stimulus, computing the phonological code, and converting it into a speech signal. Giving its specific nature, reading aloud thus has similarities and differences with both the process of (silent) reading and the process of speech production, the former being about getting from print to meaning and the latter being about getting from concepts to sounds. Since reading aloud may be construed as a print-to-sound mapping process, a key issue for such a process is the understanding of how a phonological code is translated into a sequence of articulatory gestures that correspond to the word’s sounds. Despite their importance, the operations involved in the planning and execution of articulation in reading aloud have not been investigated with the same fervor that word recognition or lexical access received. As a consequence, little empirical evidence is available on how readers perform the two steps assumed to follow, i.e., the lexical retrieval and/or the orthography-to-phonology mapping, the phonological encoding – that is the building of a sequence of well-formed phonological syllables – and the phonetic encoding – that is the computation of the phonetic-articulatory gestures of the to be uttered stimulus (Levelt et al., 1999). In most computational models of reading aloud phonological and phonetic encoding are implemented as an oversimplified set of operations (see, e.g., Rastle and Coltheart, 2000; Coltheart et al., 2001; Arciuli et al., 2010; Perry et al., 2010).

Recent empirical work has shown evidence for a double process at the level of phonological encoding in reading. Similarly to what happens in word production, reading polysyllabic words implies retrieving both segmental (i.e., word sounds) and suprasegmental information (i.e., stress) and these two types of information may be computed separately (Colombo and Zevin, 2009; Sulpizio et al., 2012a,b; Sulpizio and Job, 2013). Ascribing the computation of stress and the computation of phonemes to two separate mechanisms has important consequences on the structure of phonological and phonetic encoding since the assembling of the phonological unit will require the reader to carry out at least three operations: (a) activating the word’s segments, (b) activating the stress pattern, and (c) assembling segmental and suprasegmental information. Data on (c) are lacking, but some evidence is available for both (a) and (b).

An insight into the phonological encoding in reading has been provided by the masked onset priming effect (henceforth MOPE; Forster and Davis, 1991; see Grainger and Ferrand, 1996): target words (e.g., sink) are named faster when preceded by a masked prime with the same initial phoneme (e.g., save), than by a prime with a different initial phoneme (e.g., ball). The main account of the MOPE – the speech planning account (Kinoshita, 2000) – assumes that the effect has a serial nature and affects the segment-to-frame association (Kinoshita, 2000; Kinoshita and Woollams, 2002; Malouf and Kinoshita, 2007; Dimitropoulou et al., 2010; but see Mousikou et al., 2010). Such process allows for the active phonological segments to be assigned to an abstract frame – i.e., the word metrical frame – specifying the number of syllables and the stress pattern of the word (e.g., for the word FEcola ‘starch,’ the metrical word is ‘σ σ σ). The MOPE was also found by Schiller (2004) with a slightly different masked priming paradigm, in which participants had to read aloud Dutch words (e.g., banaan, ‘banana’) under two conditions: when preceded by a prime consisting of an onset-related word embedded in a sequence of symbols (e.g., %%balans%%, ‘balance’) and when preceded by an onset-related sequence prime that consisted of one or two letters embedded in a sequence of symbols (e.g., %%ba%%%%%%). Responses to targets were faster in both onset-related conditions than in the control, all symbols condition (%%%%%%%%) and Schiller suggested that the pre-activation of congruent phonological segments by the prime facilitates the phonological encoding of the target (see, e.g., Schiller and Kinoshita, 2007). Taken together, these findings offer support for a stage of phonological encoding in the reading system; during this stage, after having retrieved/computed word’s phonemes and stress, the reader assembles the phonological word through a rightward serial process that associates the phonological segments to a metrical frame. The resulting unit is then used to address the articulatory system (see Levelt et al., 1999 for a detailed description of the phonological encoding in speech production).

With regard to stress, some studies have investigated stress assignment to polysyllabic words addressing the question whether the computation of stress may be independent of the computation of segmental information. The results have been mixed. In a series of implicit form-priming experiments – participants first learn pairs of words (e.g., meer-water ‘lake-water’), and then had to produce the second word (e.g., water) of the pair in response to the presentation of the first (e.g., meer) – Roelofs and Meyer (1998) manipulated the stress pattern of the to be produced words (all having either the same or different stress) and did not find any stress priming effect. However, adopting different priming methodologies (all involving visible primes), some reading aloud studies have shown that the metrical structure of a word may be primed independently from its segmental content, and this is possible both when stress is assigned to pseudowords and when it is lexically retrieved (Colombo and Zevin, 2009; Sulpizio et al., 2012a,b). Possible explanations for the divergent results are offered in the General Discussion, but for the time being we assume that computation of stress and segmental information are to some extent independent. To illustrate this issue, we may refer to the Sulpizio et al.’s (2012b) study: readers were presented with prime-target word pairs that did or did not share the stress pattern (e.g., TESsera – BUfala, ‘card’ – ‘hoax’ vs. cuGIno – BUfala^1^, ‘cousin’ – ‘hoax’) and were found to be faster in reading the targets when preceded by a congruent stress prime, than when preceded by an incongruent-stress prime. The finding invites the conclusion that readers have an abstract representation of stress, quite independent from the segmental material and that the representation of stress is involved in the segment-to-frame association and in the articulatory planning of the stimulus, thus affecting target processing.

While phonemic computation and stress assignment are to some extent handled by autonomous systems, they need to interact during processing. Specifically, articulation requires a segment-to-frame association, in which the system associates the computed phonological segments to a metrical frame, and such a well-formed phonological unit will allow articulation (Dell, 1986, 1988; Levelt et al., 1999).

The speech production literature may help to shed light on the functioning of the segment-to-frame association in word reading. Since both reading aloud and speech production require the construction of a phonological unit and its conversion into articulatory programs, they share (at least in part) the stages of processing finalized to encode the phonological word and to use such a phonological word to produce the phonetic realization of the stimulus (Roelofs, 2004).

To investigate the processing of segment-to-frame association and phonological-to-phonetic mapping in word reading we run four experiments in Italian capitalizing on the fact that in such language stress is nor graphically marked neither solely determined by orthographic structure^2^ and that, therefore, any particular word’s stress pattern can only be reliably established through lexically stored information. Our results will be then generalizable to the other polysyllabic languages such as English, with a similar stress system.

Although distributional cues allow Italian readers to assign stress to pseudowords to some extent (Colombo and Zevin, 2009; Sulpizio et al., 2013), such cues play no role in word reading (Paizi et al., 2011; Sulpizio and Colombo, 2013). The fact that in Italian word stress is lexically based may be helpful to investigate phonological encoding: since there is no algorithmic procedure to assign a stress to a stimulus, the metrical structure has to be lexically retrieved and then combined with the segmental material to shape the phonological word, which will be then used by the system to address articulatory programs.

Experiments 1 and 2 investigated the MOPE and the stress priming effects by means of a masked priming paradigm, with a set of tightly controlled stimuli, trying to establish whether the two effects are facilitatory or inhibitory. Moreover, with regard to the MOPE, the use of a pure segmental prime (e.g., fe%%%% – FEcola, ‘starch’) allowed us to test whether the activation of the first phonological segments of the word automatically activates suprasegmental information as the masked segment (e.g., <fe>) might activate either a syllabic unit – which may be phonetically specified for stress (i.e., as stressed or unstressed) – or only its segmental constituents (i.e., /f/ and /e/).

We adopted the masked priming paradigm also in Experiments 3 and 4 but the aim here was to test the effect of the joint manipulation of segmental and suprasegmental information. Thus, for each prime-target pair, the prime either shared both the initial phonemes and the stress pattern with the target (e.g., FEgato – FEcola, ‘liver’ – ‘starch’), or shared the initial phonemes with the target but had a different stress pattern (e.g., feNIce – FEcola, ‘phoenix’ – ‘starch’). In the control condition, the prime-target pair shared neither segmental nor suprasegmental information, the prime being composed of a string of symbols (%%%%%%). The manipulation is particularly interesting for the fact that Italian three-syllable words have two main stress patterns (Thornton et al., 1997): antepenultimate stress (i.e., the first syllable bears stress, e.g., TAvolo ‘table’), and penultimate stress (i.e., the second syllable bears stress, e.g., coLOre ‘color’). Although their distribution differs – 80% of three-syllable words bear penultimate stress and 18% bear antepenultimate stress^3^ – reading of words bearing the dominant penultimate stress pattern is not faster, and the two patterns are assumed to be stored in the phonological lexicon (Burani and Arduino, 2004; Paizi et al., 2011). Thus, a further question we may ask is whether the prime-target manipulation affects similarly penultimate- and antepenultimate-stress targets. For the manipulation we proposed – prime-target pairs sharing both initial phonemes and stress vs. prime-target pairs sharing initial phonemes but not stress – we may sketch the following predictions: congruent primes should facilitate, and incongruent primes should inhibit, target articulation. The facilitation would be brought about by the prime pre-activating either segments and/or stress (cf. Roelofs and Meyer, 1998) congruent with the target, while in the incongruent condition the stress mismatch would be enough to delay the articulation. In fact, if we assume – according to current computational models of polysyllable word reading (Perry et al., 2010) – that readers do not start articulation until stress has been fully activated – since only determining which syllable is stressed guarantees correct performance –, we may expect that the incongruency at the suprasegmental level may be sufficient to delay the articulation, irrespective of any overlap at the segmental level. Moreover, since previous stress priming studies have shown that stress priming effects seem not to be modulated by the word stress position (Sulpizio et al., 2012b), no difference is expected between penultimate- and antepenultimate-stress targets.

## Experiment 1

In Experiment 1 we tested the MOPE in a reading aloud experiment with Italian penultimate- and antepenultimate-stress words as targets. We adopted the paradigm proposed by Schiller (2004; see also Schiller and Kinoshita, 2007), in which the target word (e.g., FEcola, ‘starch’) is preceded by an onset-related or -unrelated sequence (e.g., fe%%%%; mi%%%%). In this way, we are able to exclude any effect of suprasegmental material that, in case of a whole word prime (as, e.g., FEgato ‘liver’), might be elicited by the activation of stress information. In addition, in order to establish the direction of the effect we also included a control condition that did not involve orthographic information.

The aim of Experiment 1, however, was not only to replicate previous studies showing that onset-related primes facilitate the computation of target phonology during reading aloud, but also to test whether a pure segmental prime may also activate suprasegmental information. In the onset-related condition, prime and target shared the first syllable as they were segmentally identical; however, the target syllable was either stressed (e.g., FEcola ‘starch’) or unstressed (e.g., feNIce ‘phoenix’), and thus the prime syllable could or could not be congruent with the target first syllable for stress pattern. This allows us to propose two alternative predictions: first, if the prime affects an abstract phonological level of computation, such as the segment-to-frame association, then readers should be faster reading a target word in the onset-related condition than in either the onset-unrelated condition or the control condition (Schiller, 2004), and this should be true for both antepenultimate and penultimate stress words. Alternatively, if the prime affects the phonetic level of target computation – by activating a phonetic syllabic unit containing also information about stress – then we should expect different results for penultimate- and antepenultimate-stress targets. The reason for this is that penultimate-stress targets start with an unstressed syllable whereas antepenultimate-stress targets start with a stressed syllable. Thus, if the prime activates a stressed syllable, it might facilitate antepenultimate-, but not penultimate-stress targets; differently, if the prime activates an unstressed syllable, it might facilitate penultimate-, but not antepenultimate-stress targets.

### Method

#### Participants

Twenty-four students (six males, mean age: 23.33; *SD*: 4.73) from the University of Trento took part in the experiment. They received course credit for their participation. All participants were Italian native speakers with normal or corrected-to-normal vision. This and all the following experiments were carried out in accordance with the recommendations of the University of Trento ethics committee.

#### Materials

Targets were two sets of 24 three-syllable words each. One set comprised penultimate-stress words and the other antepenultimate-stress words. Words were selected from the CoLFIS database (Bertinetto et al., 2005) and were matched on: frequency, orthographic neighborhood size, orthographic neighbors’ summed frequency, and bigram frequency (**Table 1**). Words in the two sets were also matched on their first syllable, i.e., for each word in a set there was a word in the other set starting with the same syllable as, e.g., FEcola ‘starch’ and feRIta ‘wound.’ All words were six letters long and had the same CVCVCV syllabic structure. All stimuli are listed in the Appendix.

**Table 1:** Summary statistics: mean (and standard deviation) for target words used in Experiments 1–3.

	Stress type	
Variables	Antepenultimate	Penultimate
Word frequency	10.58 (17.42)	10.7 (17.63)
N of orthographic neighbors	3.66 (2.38)	3.29 (2.27)
Neighbors’ frequency	25.38 (60.46)	17.49 (24.98)
Bigram frequency	11.33 (0.48)	11.31 (0.34)

*Word frequency measures are calculated out of one million occurrences (Bertinetto et al., 2005); bigram frequency is log transformed on the basis of the natural logarithm.*

Each target (e.g., FEcola ‘starch’) was preceded by three different primes: (i) a control condition, in which the prime consisted of a string of symbols (%%%%%%); (ii) an onset-related condition, in which prime and target share the first syllable (e.g., fe%%%%); (iii) an onset-unrelated condition, in which prime and target differ in the first syllable (e.g., mi%%%%). Three different lists were created, and each target appeared once in each list in a different prime condition. Within each list the three prime conditions appeared the same number of times.

#### Procedure

Participants were tested individually. They were instructed to read the targets aloud as quickly and accurately as possible. No information was given about the presence of the primes, which was revealed only after the experiment.

The experiment was run using E-Prime software (Psychology Software Tools, Pittsburgh, PA, USA). Each target started with a fixation cross, in the center of the screen, for 400 ms. The fixation cross was followed by a forward mask of hash marks (#), which was displayed for 500 ms in the center of the screen. The prime was then presented for 50 ms in lower-case letter, in the same location, followed by the target word, displayed in upper-case letters in the same position as the prime. The target remained on the screen until the participant began to read or for a maximum of 1,500 ms. A voice key connected to the computer measured reaction times (RTs) in ms from the onset of pronunciation.

The inter-stimulus interval was 1,500 ms. A short practice session preceded the experiment.

Each participant received all three lists, each list in a separate block separated by a short interval. Each block contained only one token of target and an equal number of the three prime-target pairs; the order of blocks was counterbalanced across participants and the order of prime-target pairs was randomized within each block. The experimenter noted the naming errors or apparatus failures on the fly.

### Results

**FIGURE 1:**
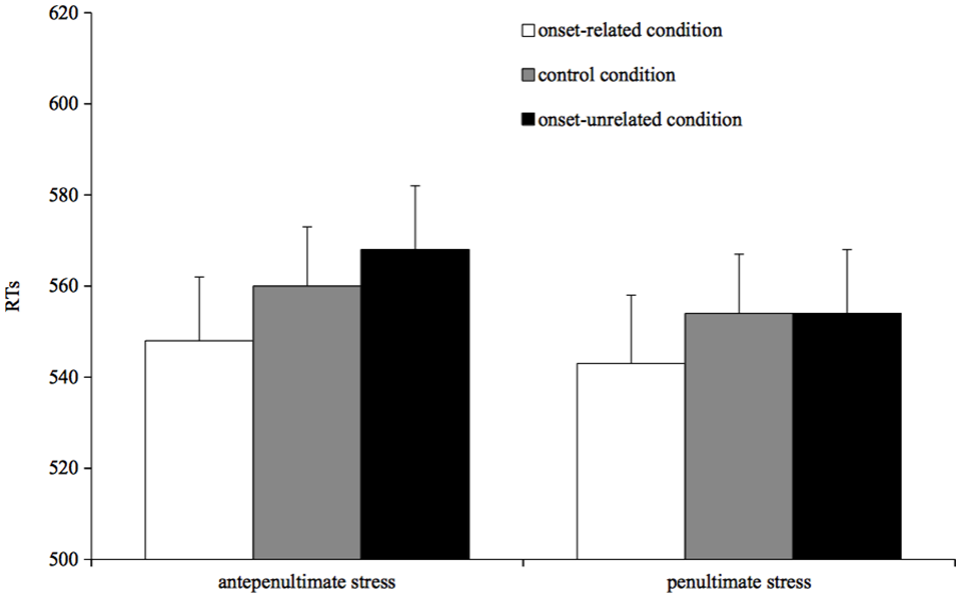
**Mean reading times for correct responses by condition in Experiment 1**.

Responses shorter than 200 ms and invalid trials due to technical failures accounted for 1.3% of all data points and were discarded from the analyses; outliers (0.9% of all data points) were identified and removed following the Van Selst and Jolicoeur’s (1994) procedure. Three items (PAtina ‘patina,’ coLEra ‘cholera,’ Mitilo ‘mussel,’ all above 30% of errors) were also excluded from analyses due to the very high percentage of errors participants made. Naming errors were few (2.4% of all data points) and were not analyzed. Naming times were analyzed using mixed-effects models (Baayen et al., 2008). The models were fitted using the lmer function in R software. The models included prime type (related, unrelated, and control) and stress of the target (penultimate and antepenultimate) as fixed factors^4^. For the random factors, a maximal random structure approach was used (by participants and by items random intercepts and slopes; see Barr et al., 2013). The analysis started with a full factorial model including the main effects and the two-way interaction. The model was progressively simplified by removing the variables that did not significantly contribute to the goodness of fit of the model. Variables were evaluated one by one on the basis of likelihood ratio tests: those whose exclusion did not decrease significantly the model goodness of fit were removed from the analysis. Statistics of the best model are reported. Statistical significance of the fixed parameters was evaluated using the MCMC procedure, sampling 10,000 times (Baayen et al., 2008). Results are reported in **Figure 1**.

The full factorial model revealed that the prime type by stress of the target interaction was not significant, and it was dropped from the analysis as it did not significantly increase the model goodness of fit (χ^2^ = 2.47, *p* > 0.2). The reduced model revealed that prime type significantly affected reading of target words, with slower reading times for targets preceded either by unrelated primes (β = 14.46, *SE* = 3.37, *t* = 3.61, *p* < 0.001) or by control primes (β = 10.01, *SE* = 3.61, *t* = 2.76, *p* = 0.005) than for targets preceded by related primes. The unrelated and the control conditions did not differ (*t* = 1.22, *p* > 0.2). The effect of target stress was not significant (*t* = -1.25, *p* > 0.2).

### Discussion

The results of Experiment 1 show a clear effect of the segmental overlap on reading times: readers were facilitated in reading a target word in the onset-related condition in comparison to both the onset-unrelated and the control condition. The pattern goes in the same direction for penultimate- and antepenultimate-stress targets, suggesting similar processing in the computation of segmental information for both types of words.

The pattern we obtained is entirely compatible with Schiller’s (2004; Schiller and Kinoshita, 2007) explanation: in the onset-related condition the prime pre-activates the initial phonological segments of the target at the level of phonological encoding. According to such a view, the active units are phonological segments and not phonetically specified syllabic units.

The analogous pattern obtained for penultimate- and antepenultimate-stress target supports this claim. In our experiment, the congruent prime always coincided with the first syllable of the target and, thus, the prime might have activated a syllabic unit rather than two phonological segments. However, in Italian a syllabic unit is realized in one of two different phonetic versions, i.e., as stressed or unstressed. Thus, the prime could have affected the target at a phonetic level, by activating a phonetically specified syllabic unit, which would also activate information about stress. This being the case, a different pattern for penultimate- and antepenultimate-stress targets would be expected since pre-activation of stressed syllables would facilitate reading antepenultimate-stress targets (which start with a stressed syllable) but not penultimate-stress targets (which start with an unstressed unit) and pre-activation of unstressed syllables would lead to the opposite pattern. The results of our experiment showing a parallel pattern for both penultimate- and antepenultimate-stress words suggest that the prime exerts its effect at an abstract phonological level, with a benefit for onset-overlapping targets during the word phonological encoding (Schiller, 2004)^5^.

In Experiment 2 we investigated the effect of suprasegmental priming on the phonological encoding of the word using the same set of target words of Experiment 1.

## Experiment 2

The aim of the present experiment was to establish whether the masked stress priming is effective in generating a stress priming effect, and whether such an effect is facilitatory or inhibitory in nature. The stress priming effect reported by previous studies has never been tested against a control condition (Colombo and Zevin, 2009; Sulpizio et al., 2012a,b), with the consequence that it is still unclear whether priming the metrical structure of a word facilitates or inhibits reading it aloud. Moreover since all aforementioned studies adopted a visible priming technique – in which readers explicitly processed the prime – it cannot be excluded that the effect of stress priming they reported may have a strategic component. To rule out this hypothesis, we used the masked priming paradigm with prime-target pairs that differed at the segmental level but did or did not share the metrical structure. In this way, we would be able to assess whether primes sharing or not sharing stress with the targets (i.e., the congruent vs. incongruent condition) affect target reading, with respect to a non-linguistic control condition, over and above any effect due to the prime and target mismatch at the segmental level.

### Method

#### Participants

Twenty-four student (four males, mean age: 20.26; *SD*: 1.99) from the University of Trento took part in the experiment. They received course credit for their participation. All participants were Italian native speakers with normal or corrected-to-normal vision.

#### Materials

The same target words of Experiment 1 were used. Prime words had the same syllabic length and structure of the targets. Penultimate- and antepenultimate-stress prime words were matched on frequency, orthographic neighborhood size, orthographic neighbors’ summed frequency, and bigram frequency (**Table 2**). All stimuli are listed in the Appendix. Primes and targets were paired in such a way as to obtain three prime conditions for each targets: a stress congruent condition, with prime and target sharing the same stress pattern (e.g., CInema – FEcola, ‘cinema’ – ‘starch’); a stress incongruent condition, with prime and target bearing a different stress (e.g., caNAle– FEcola, ‘channel’ – ‘starch’); and a control condition, in which the target word was preceded by a string of symbols (e.g., %%%%%% – FEcola, ‘starch’). Primes and targets were not semantically related and never shared the initial syllable.

**Table 2:** Summary statistics: mean (and standard deviation) for prime words used in Experiments 2 and 3.

	Stress type	
Variables	Antepenultimate	Penultimate
Word frequency	48.41 (105.33)	44.08 (59.39)
N of orthographic neighbors	3.37 (2.08)	3.41 (2.16)
Neighbors’ frequency	15.26 (21.98)	29.82 (38.11)
Bigram frequency	11.3 (0.33)	11.42 (0.39)

*Word frequency measures are calculated out of one million occurrences (Bertinetto et al., 2005); bigram frequency is log transformed on the basis of the natural logarithm.*

#### Procedure

The same procedure as in Experiment 1 was adopted.

### Results

Responses shorter than 200 ms or longer than 1500 ms as well as invalid trials due to technical failures accounted for the 2.6% of all data points and were discarded from the analyses; outliers (1% of all data points) were identified and removed using the Van Selst and Jolicoeur’s (1994) procedure. Due to the high number of errors, two items (PAtina ‘patina,’ Mitilo ‘mussel,’ above 30% of errors) were excluded from analyses. Naming errors were few (2.5%) and were not analyzed.

**FIGURE 2:**
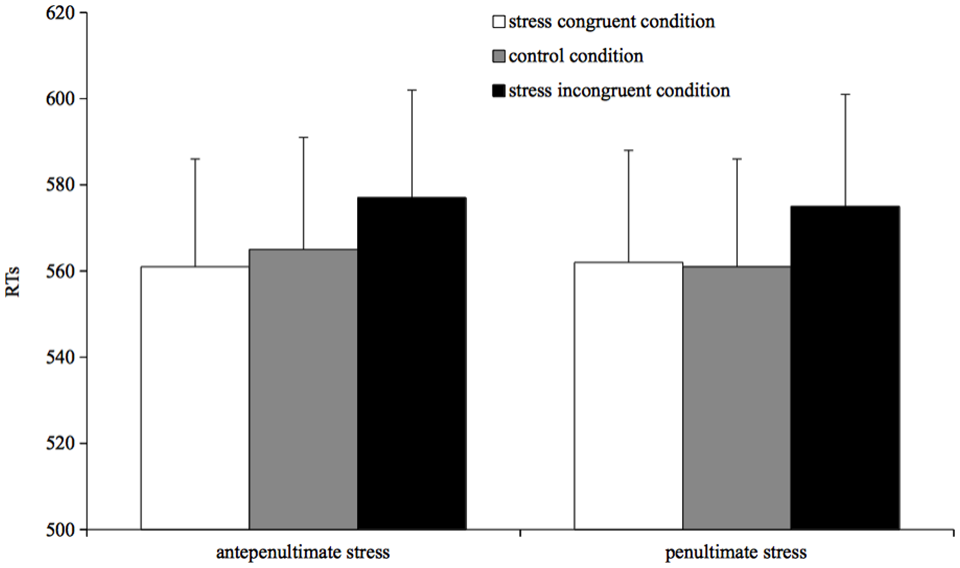
**Mean reading times for correct responses by condition in Experiment 2**.

Naming times were analyzed using mixed-effects models (Baayen et al., 2008). Results are reported in **Figure 2**.

The model was run with RTs as dependent variable and prime type (congruent stress, incongruent stress, and control) and stress target (penultimate and antepenultimate) as fixed factors.

The full factorial model revealed that the prime type by stress target interaction was not significant, and as it did not significantly increase the model goodness of fit (χ^2^ < 1) it was dropped from the analysis. The simplified model showed that prime type significantly affected target reading times: participants were slower when reading targets preceded by incongruent stress primes than preceded by both congruent stress primes (β = 10.49, *SE* = 4.47, *t* = 2.34, *p* = 0.01) and control primes (β = 9.19, *SE* = 4.51, *t* = 1.91, *p* = 0.05) No difference was found between targets preceded by congruent stress primes and by control primes (*t* < 1). No main effect of stress target was found (*t* < 1).

### Discussion

The pattern shown by the analyses of reading times is clear: readers are slower when reading a target word preceded by a prime bearing a different stress pattern than a target preceded by a prime bearing the same stress pattern or by a control prime. Moreover, the stress prime effect is not affected by the type of word stress pattern as revealed by the absence of a prime type by stress type interaction.

The results of Experiment 2 replicate findings on stress priming reported previously (Sulpizio et al., 2012b), but they add new insights about the computation of stress in reading. In particular, the finding of a stress priming effect when the prime is masked not only corroborates the view that the metrical structure of a word may be primed independently from its segmental content, but also suggests that the word stress pattern is automatically activated by lexical computation as well as by segmental phonological information.

With regard to the nature of the priming effect, our results show that target words preceded by stress-incongruent primes were read more slowly than those preceded by stress-congruent primes, and this was true for both penultimate- and antepenultimate-stress targets. Thus, the findings extend Sulpizio et al.’s (2012b) results by showing that the stress priming effect on naming times is automatic, i.e., it is not driven by strategic mechanisms, since it emerges also when readers are not aware of the presence of primes. As previous works suggest (Colombo and Zevin, 2009; Sulpizio et al., 2012b), the locus for the stress priming effect is the stage of phonological encoding.

Note that the prime-target pairs of Experiment 2 always differed at the segmental level. It might be argued that such segmental mismatch might have contributed to the pattern we found, as the phonological segments activated by the prime could have interfered with the selection of the segments of the target. However, if that were the case, the effect of segmental mismatch would have been visible also in the congruent-stress condition, with slower reading times than the control condition, which is clearly not the case. The absence of segmental inhibition is also in line with the results of Experiment 1, where there was no difference between targets in the control and in the segmentally incongruent condition, and reinforces the idea that, under our experimental conditions, segmental information may facilitate but does not hinder word processing (e.g., Schiller and Kinoshita, 2007).

Taken together, the results of Experiments 1 and 2 show an interesting asymmetry: a segmental prime without stress information speeds up the reading of a segmentally consistent target; a stress prime, keeping segmental information (incongruently) constant, slows down the reading of stress inconsistent targets. This is *prima facie* evidence that segmental and suprasegmental information affect word reading independently and with an opposite pattern. Moreover, in both Experiments 1 and 2 antepenultimate- and penultimate-stress targets were similarly affected by segmental and suprasegmental priming; following previous research, both the facilitation for prime-target segmental overlapping pairs and the inhibition for prime-target incongruent stress pairs may be located at the level of phonological output buffer when the segment-to-frame association takes place (Kinoshita, 2000; Sulpizio et al., 2012b).

In Experiments 3 and 4 we further tested how readers encode the phonological word by jointly manipulating the overlap between stress and phonemes in prime/targets pairs. To our knowledge, this issue has never been investigated in the reading literature, in spite of being crucial for any model of polysyllabic word reading.

## Experiment 3

In this experiment we investigated the processes of segment-to-frame association by directly testing how readers assemble the phonological segments with the stress metrical structure of the word they have to produce. We used the same target words of the two previous experiments, and varied the degree of overlap of segmental and suprasegmental information between primes and targets. To illustrate, each target (e.g., FEcola, ‘starch’) could be preceded by: (a) a congruent prime, in which prime and target shared both the first syllable and the stress pattern (e.g., FEgato, ‘liver’); (b) an incongruent prime, in which prime and target shared the first syllable but not the stress pattern (e.g., feNIce, ‘phoenix’); (c) a control prime, i.e., a sequence of symbols (%%%%%%). The incongruent prime condition is the critical one. In fact, although both congruent and incongruent primes would cause pre-activation of the segmental level, in the latter case the pre-activated phonemes might not be associated to the correct metrical frame until the stress pattern has been identified (cf. Perry et al., 2010). This would interfere with the segment-to-frame association and with the processes occurring further down stream by delaying the selection of the correct metrical frame and its association with the phonological segments and the planning of articulation. No such delay would occur in the congruent prime condition, where the pre-activation of both the initial phonemes and the correct stress pattern would speed up articulation.

### Method

#### Participants

Twenty-four students (11 males, mean age: 28, *SD*: 5.57) took part in the experiment. None participated to both experiments. Participants were all from the University of Trento and received course credit for their participation. All participants were Italian native speakers with normal or corrected-to-normal vision.

#### Materials

The same target and prime words of Experiment 2 were used. However, the pairing of primes and targets was modified in order to obtain three conditions: 16 prime-target pairs sharing both the initial syllable and the stress pattern (e.g., FEgato– FEcola, ‘liver’ – ‘starch’); 16 pairs sharing the same initial syllable but having a different stress pattern (e.g., feNIce – FEcola, ‘phoenix’ – ‘starch’), and 16 pairs not sharing either segmental or stress information (control condition; e.g., %%%%%% – FEcola, ‘starch’). Primes and targets were never semantically related. Three different lists were created, so that each target appeared only once in each list in a different prime condition. Within each list the three prime conditions appeared the same number of times.

#### Procedure

The same procedure as in Experiment 1.

### Results

Responses shorter than 200 ms or longer than 1500 ms as well as invalid trials due to technical failures accounted for the 2.2% of all data points and were discarded from the analyses; outliers (2.5% of all data points) were also removed using the Van Selst and Jolicoeur’s (1994) procedure. Due to its high number of error (above 30%), one item (PAtina ‘patina’) was removed and not further considered in the analyses. Naming errors were few (2.9%) and were not analyzed.

**FIGURE 3:**
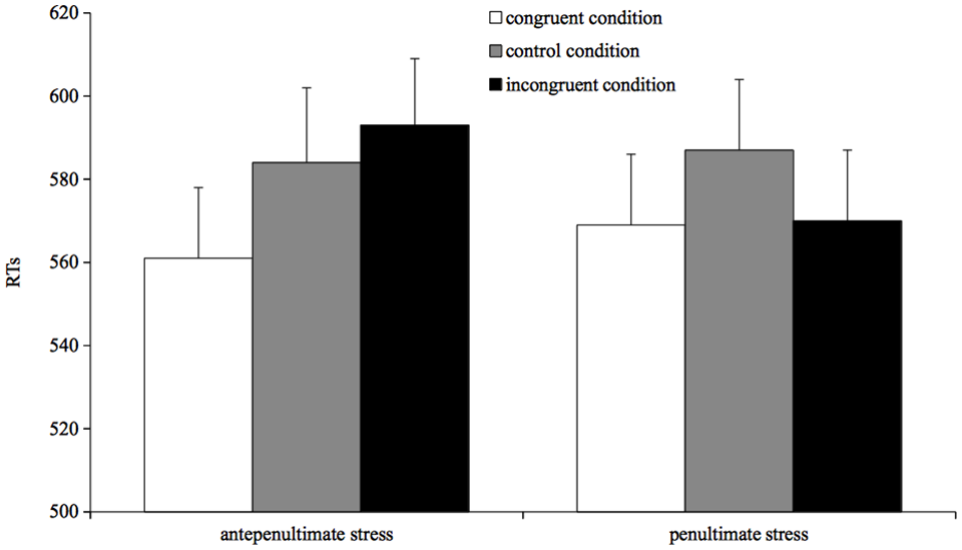
**Mean reading times for correct responses by condition in Experiment 3**.

Naming times were analyzed using mixed-effects models (Baayen et al., 2008). Results are reported in **Figure 3**.

The model was run with RTs as dependent variable and prime type (congruent, incongruent, and control) and stress target (penultimate and antepenultimate) as fixed factors. The prime type by stress target interaction was significant (β = -14.03, *SE* = 6.78, *t* = -2.10, *p* = 0.03), showing that the three primes affected antepenultimate- and penultimate-stress targets differently. Direct comparisons between conditions were assessed through separate analyses on the two types of targets. For antepenultimate-stress targets, the model showed that participants were faster in reading targets when preceded by congruent primes than when preceded by either incongruent primes (β = 17.58, *SE* = 4.88, *t* = 3.59, *p* < 0.001) or control primes (β = 11.94, *SE* = 4.92, *t* = 2.42, *p* = 0.01). Incongruent and control conditions did not differ from each other (*t* = 1.13, *p* > 0.2). A different pattern was found for penultimate-stress words: participants were faster in reading a penultimate-stress target when preceded by a congruent prime than when preceded by a control prime (β = 14.92, *SE* = 4.68, *t* = 3.18, *p* = 0.001); surprisingly, participants were also faster in reading a target when preceded by an incongruent prime than when preceded by a control prime (β = 15.35, *SE* = 4.69, *t* = 3.26, *p* = 0.001). No difference was found between congruent and incongruent prime condition (*t* < 1).

To sum up, the effect of the incongruent prime condition on naming speed appears to be asymmetric: it does not differ from the control condition for antepenultimate-stress targets, but it is facilitatory for penultimate-stress targets.

### Discussion

The results of Experiment 3 show that penultimate- and antepenultimate-stress targets are processed more rapidly in the congruent than in the control condition. However, the two types of targets differ in the incongruent prime condition: reading times to incongruent antepenultimate-stress targets and to control targets do not differ, and both are read more slowly than congruent antepenultimates-stress targets; however, incongruent penultimate-stress targets are read as quickly as congruent penultimate-stress targets and both are processed more rapidly than control targets. Thus, the incongruent prime condition hinders responses to antepenultimate-stress targets but does not affect penultimate-stress targets.

The results for penultimate stress targets show that the overlap of segmental information between prime and target is sufficient to drive a process for incongruent targets that is quantitatively analogous to that driven by the congruent condition, in which primes and targets overlap for suprasegmental as well as segmental information. This result for penultimate-stress words is not only sharply different from the pattern obtained for antepenultimate-stress words in the same condition, but it is also quite in contrast with the pattern obtained in Experiment 2, where slower reading times were obtained for incongruent pairs when prime-target pairs had different stress but were also entirely different at the segmental level.

For antepenultimate-stress targets, however, the data pattern differently: targets in the incongruent prime condition are read more slowly than targets in the congruent prime condition. To understand such pattern we may look at the first two experiments, which show that the prime-target segmental congruency speeds up responses (Experiment 1), whereas the prime-target suprasegmental incongruency slows down the reading times (Experiment 2). Thus, in Experiment 3, in which the two factors were jointly manipulated, the actual pattern for the incongruent condition could be the outcome of the combination of the segmental overlap and the suprasegmental mismatch. Specifically, segmental match speeds up frame to segment association, but the concurrent presence of incongruent stress information slows down such process, with the result that the two effects cancel out. Thus, the crucial aspect becomes how the system incorporates congruent and incongruent segmental and suprasegmental information in time.

The asymmetry between antepenultimate- and penultimate-stress targets we obtained for the incongruent condition in Experiment 3 lends itself to several possible interpretations. The processing account we provide below seems to us to be both empirically consistent and theoretically valid, but further data will be necessary to rule out alternative accounts.

We ascribe the asymmetric pattern to the operations that take place at the level of phonological output buffer, where lexical and sub-lexical routes converge and the system pools together the information coming from the two routes to drive the stimulus pronunciation; in our view, the buffer comprises a system for phonemic activation and one for stress assignment (for a similar proposal, see Perry et al., 2010). Within the phonological output buffer, we assume that the segment-to-frame association – i.e., the association of phonemes to a metrical frame – and the phonological-to-phonetic mapping – i.e., the mapping of abstract linguistic information into motor commands – take place rightward incrementally (cf. Kinoshita, 2000). Thus, for the first syllable of three-syllable antepenultimate-stress words there is activation of both its phonemes and the stress pattern while for the first syllable of penultimate-stress words there is activation of its phonemes while it is the second syllable that requires the activation of both its phonemes and the stress pattern. Accordingly, we assume that, during the segment-to-frame association, the stress system specifies the tonic syllable among the available segmental material: specifically, at the level of phonological output buffer, once enough evidence (coming from lexical and sub-lexical route) for a stress pattern is available, the stress system specifies which syllable should be articulatory implemented as stressed. Note that for reading there may not be the need to specify information about the number of syllables, as in word reading, the number of syllables and their internal organization may be inferred by orthography, with the system able to arrange the identified letters (or group of letters) into a graphosyllabic representation (see, e.g., Caramazza and Miceli, 1990; Perry et al., 2007, 2010; see also Chetail et al., 2014 for evidence that the structure of a letter string can be determined simply on the basis of consonant and vowel identification). Furthermore, we assume that the reading system starts the planning of articulation as soon as the relevant information for the to-be-planned unit is active. We may call this the use-information-as-soon-as-possible (UIASAP) approach. That is to say, within the phonological output buffer, as soon as usable information becomes available it is incorporated in the open frame the system builds for the phonetic encoding of the stimulus, which then addresses the motor programs to execute articulation. This would yield different patterns for antepenultimate- and penultimate-stress targets in the incongruent condition: for the former, articulation may start as soon as the first syllable is encoded, since both segmental and suprasegmental information is already available; for the latter, articulation needs to wait up to the second syllable since information about stress becomes available at that point. The inconsistent stress prime would differently affect the two types of words as a function of the unit to be processed. For antepenultimate-stress words the interference would be stronger as it would impact on the to-be-articulated syllable. For penultimate-stress words, however, there would be time to mitigate the impact of the incongruent stress prime since articulation cannot start until the information about stress becomes available on the second syllable; this being the case, the system might capitalize on the available segmental information, which is not affected by the suprasegmental-stress mismatch, performing in a similar manner for targets with congruent- and incongruent primes.

An alternative explanation of our results may rest on the distributional asymmetry of the two stress patterns. In Italian, 80% of three-syllable words bears penultimate stress while 18% bears antepenultimate (Thornton et al., 1997), and it might be argued that the penultimate stress pattern would work as a default pattern (Colombo, 1992). Thus, penultimate stress would reach the activation level quite easily, with low chance to be interfered with by any other pre-activated, less frequent stress pattern. The antepenultimate-stress pattern would show the opposite picture, as it is less represented in the lexicon and it would need a high activation level to be selected; as a consequence, it would have a high probability to be interfered with by the partial activation of the penultimate-stress (default) pattern. According to this distributional view, the asymmetry we found for penultimate- and antepenultimate-stress targets in Experiment 3 would be fully accountable for by the different weight the two stress patterns have in the reading system, the former being the default, more available pattern.

The Italian lexicon offers a good test to adjudicate between the UIASAP and the distributional pattern hypotheses, i.e., three-syllable words with final stress, which is the least frequent stress patterns (around 2% of three-syllable words). Thus, in Experiment 4 we performed a critical test of the two alternative accounts by using final-stress words as targets, i.e., words bearing stress on the last syllable, which is orthographically marked (e.g., coliBRÌ, hummingbird). Note that for these words the suprasegmental information may be computed sub-lexically, as the accent mark may directly activate the corresponding stress pattern. However, as in other domains of orthographic processing, we think that the system always engages in lexical as well as non-lexical processing (see, e.g., Peressotti et al., 2003). Thus, we think that final-stress words are a very good test for the UIASAP hypothesis.

The distributional pattern and the UIASAP hypothesis make opposite predictions about the pattern the final-stress words should elicit. If the different pattern of results found for antepenutlimate- and penultimate-stress words is due to their distributional properties, then we expect final-stress words to behave as the antepenultimate-stress words, since both are rare patterns in the language. This being the case, for antepenultimate-stress and final-stress words we expect both incongruent and control condition to be slower than the congruent condition, and not to differ from each other. On the other hand, if the difference between antepenultimate- and penultimate-stress pattern is due to left-to-right processing, as assumed by the UIASAP proposal, then we may expect the final-stress words to pattern with the penultimate-stress words. In particular, antepenultimate-stress words should show slower reading times in the incongruent than in the congruent condition, while both penultimate-stress and final-stress words should show similar reading times in the congruent and incongruent condition due to the earlier availability of the segmental information and to the absence of mismatching stress information on the first syllable that allows for the articulation of the word to begin.

In Experiment 4 we used a new set of stimuli comprising final-stress words as well as new penultimate- and antepenultimate-stress words. The aim of the experiment is twofold: first, to test for the replicability and robustness of the effect we found in Experiment 3; second, to adjudicate between the UIASAP and the distributional pattern hypotheses.

## Experiment 4

### Method

#### Participants

Twenty student (five males, mean age: 23.7; *SD*: 4.9) from the University of Trento took part in the experiment. They received course credit for their participation. All participants were Italian native speakers with normal or corrected-to-normal vision.

#### Materials

Three sets of three-syllable words were selected as targets. One set included final-stress words, one set penultimate-stress words, and one set antepenultimate-stress words. No stimulus was a target in any of the previous experiments. Of the 56 targets, half were final-stress words (mean frequency: 79.85 occurrence per million) and the other half was equally divided between penultimate- and antepenultimate-stress words (mean frequency: 100.71 and 43.50 occurrence per million, respectively). Words were selected from the CoLFIS database (Bertinetto et al., 2005). The final stress targets included 19 words of six letters and nine words of seven letters. The penultimate- and antepenultimate-stress words were all six letters in length and had a CVCVCV syllabic structure.

As in Experiment 3, for each target (e.g., REsina, ‘resin’) there were three primes: (i) a word sharing the initial syllable and the stress pattern with the target (e.g., REgola, ‘rule’); (ii) a word sharing the initial syllable but not the stress pattern with the target (e.g., reGIme, ‘regime’); (iii) a string of symbols (%%%%%%). In condition, the incongruent-stress condition, primes for final-stress targets were either penultimate-stress words (14/28) or antepenultimate-stress words (14/28), for penultimate-stress targets primes were antepenultimate-stress words, and for antepenultimate-stress targets primes were penultimate-stress words.

The sets of prime words were matched on: frequency, orthographic neighborhood size, orthographic neighbors’ summed frequency, bigram frequency (**Table 3**). Primes and targets were not semantically related. All stimuli are listed in the Appendix. Three different lists were created, with each target appearing only in one list in a different prime condition.

**Table 3:** Summary statistics: mean (and standard deviation) for prime words used in Experiment 4.

	Antepenultimate stress targets		Penultimate stress targets		Final stress targets	
Variables	Congruent stress prime	Incongruent stress prime	Congruent stress prime	Incongruent stress prime	Congruent stress prime	Incongruent stress prime
Word frequency	73.57 (121.41)	58 (82.31)	96.42 (129.4)	91.07 (159.72)	2.35 (3.58)	3.64 (7.01)
Letters length	6 (0)	6 (0)	6 (0)	6 (0)	6.32 (0.61)	6.46 (0.69)
N of orthographic neighbors	3.14 (1.91)	4 (1.88)	3.92 (2.52)	3.14 (1.4)	2.42 (1.23)	2.96 (1.62)
Neighbors’ frequency	43.41 (67.31)	15.35 (14.29)	30.69 (40.3)	32.91 (29.47)	14.95 (27.53)	31.34 (61.87)
Bigram frequency	11.74 (0.15)	11.79 (0.32)	11.7 (0.24)	11.71 (0.3)	11.65 (0.28)	11.62 (0.31)

*Word frequency measures are calculated out of one million occurrences (Bertinetto et al., 2005); bigram frequency is log transformed on the basis of the natural logarithm.*

#### Procedure

The same procedure as in Experiment 1 was adopted.

### Results

Responses shorter than 200 ms or longer than 1500 ms as well as invalid trials due to technical failures accounted for the 2.9% of all data points and were discarded from the analyses; outliers (0.3% of all data points) were also removed using the Van Selst and Jolicoeur’s (1994) procedure.

Participants did few naming errors (2.7% all data points) and were not analyzed.

**FIGURE 4:**
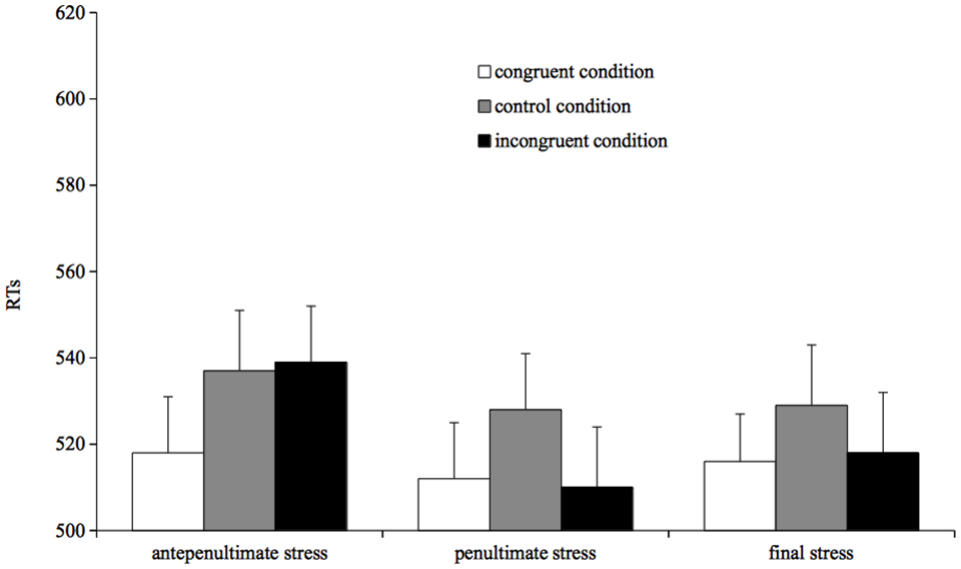
**Mean reading times for correct responses by condition in Experiment 4**.

Naming times were analyzed using mixed-effects models (Baayen et al., 2008). Results are reported in **Figure 4**.

The full factorial model was run with RTs as dependent variable and prime type (congruent, incongruent, and control) and stress target (antepenultimate, penultimate, and final stress) as fixed factors. The model showed that prime type and stress target interacted, and that the effect of prime type on antepenultimate-stress targets differed from that the prime type had on both penultimate- (β = -18.44, *SE* = 9.39, *t* = -1.96, *p* = 0.04) and final-stress targets (β = -16.11, *SE* = 8.18, *t* = -1.96, *p* = 0.04). No effect of stress target was reported for the control condition (antepenultimate vs. penultimate: *t* < 1; antepenultimate vs. final: *t* < 1; penultimate vs. final: *t* < 1). To further explore the interaction, we run separate analyses on the three types of targets.

#### Antepenultimate Stress Targets

The pattern parallels that of Experiment 3. Specifically, participants were faster in reading a target preceded by congruent primes than preceded by a control primes (β = 19.18, *SE* = 6.73, *t* = 2.84, *p* = 0.004), and were faster in reading a target preceded by a congruent prime than preceded by an incongruent prime (β = 17.68, *SE* = 6.86, *t* = 2.57, *p* = 0.01). The incongruent prime and the control prime condition did not differ from each other (*t* < 1).

#### Penultimate Stress Targets

Again, the pattern parallels that of Experiment 3: participants were faster in reading a target preceded by a congruent prime than preceded by a control prime (β = 14.36, *SE* = 6.33, *t* = 2.26, *p* = 0.02), and were faster in reading a target preceded by an incongruent prime than preceded by a control prime (β = 14.97, *SE* = 6.40, *t* = 2.33, *p* = 0.01). No difference was found between the congruent and incongruent prime condition (*t* < 1).

#### Final Stress Targets

Participants were faster in reading a target preceded by a congruent prime than preceded by a control prime (β = 10.38, *SE* = 4.65, *t* = 2.23, *p* = 0.02), and were faster in reading a target preceded by an incongruent prime than preceded by a control prime (β = 8.54, *SE* = 4.66, *t* = 1.83, *p* = 0.06). No difference was found between congruent and incongruent prime condition (*t* < 1).

### Discussion

The results of Experiment 4 are straightforward: for penultimate- and antepenultimate-stress targets we replicated the pattern found in Experiment 3, with a facilitation for both congruent and incongruent targets for penultimate-stress words, and a dissociation between congruent and incongruent targets for antepenultimate-stress words, the former being faster than the control and the latter being as slow as the control. This pattern strengthens the results of Experiments 3 generalizing them to a new set of stimuli.

The novel result is that final-stress targets show a pattern identical to penultimate-stress words, that is to say they show similar reading times for both stress congruent and stress incongruent targets (both faster than the control condition). The analogous pattern found for penultimate- and final-stress targets suggests that the difference between antepenultimate-stress words, on the one hand, and penultimate- and final-stress words, on the other hand, is not a consequence of distributional differences among the stress patterns, the latter (final stress) being much less frequent than the former (with 2% of words bearing final stress vs. 18% of words bearing antepenultimate stress). Instead, the asymmetry in the incongruent condition is more consistent with the UIASAP proposal that ascribes the difference to the way in which the operations within the phonological output buffer take place during reading aloud.

## General Discussion

In four reading aloud experiments, using a masked priming paradigm, we investigated the timing of the operations that occur in the phonological output buffer in order for readers to assemble segmental and suprasegmental information for articulating the word phonological form. Across experiments, we manipulated the degree of segmental and suprasegmental overlap between prime-target pairs of three-syllable Italian words varying in stress position. The results shed new light on several issues relevant for the understanding of how the stage of segment-to-frame association and phonological-to-phonetic mapping takes place in polysyllabic word reading.

The effect of segmental overlap we found in Experiment 1 is a robust, often replicated effect (see, e.g., Kinoshita, 2000; Schiller, 2004; Malouf and Kinoshita, 2007; Schiller and Kinoshita, 2007; Dimitropoulou et al., 2010). Since the effect emerges for orthographically dissimilar but phonologically similar prime-target pairs, and not for phonologically dissimilar but orthographically similar prime-target pairs, it has been ascribed to the stage of phonological encoding: an onset-congruent prime speeds up target reading by facilitating the segment-to-frame association process, which proceeds rightward incrementally and may thus benefit from a segmental phonological pre-activation occurring at the beginning of the word (Schiller, 2004). The results of Experiment 1 are consistent with the previous studies and provide further evidence that the prime onset overlap affects target reading at an abstract phonological level, before the articulatory programs are addressed. The claim follows from the fact that the same segmental prime (e.g., fe%%%%) affected equally penultimate-stress targets (e.g., feRIta, ‘wound’) – whose first syllable is unstressed – and antepenultimate-stress targets (e.g., FEcola, ‘starch’) – whose first syllable is stressed. As syllables may be phonetically implemented as either stressed or unstressed, the implication is that the segmental prime activates the graphemes up to their phonological representation without specifying any phonetic detail. In fact, by using primes consisting of two graphemes (letters), wholly overlapping or wholly not overlapping with the first syllable of the target, followed by a sequence of %, we were effective in withholding information about lexical stress from the prime (i.e., whether a word bears penultimate or antepenultimate stress), but we also withholded information about syllabic stress (i.e., whether, at the articulatory level, a syllable has to be implemented as stressed or not). Had the computation of the prime proceeded up to the phonetic encoding, the prime segments should have activated either the stressed or the unstressed version of the corresponding syllabic unit, and thus different results should have been expected for penultimate- and antepenultimate-stress targets, with e.g., an unstressed syllable facilitating the reading of penultimate-stress targets (which start with an unstressed syllable), but not the reading of antepenultimate-stress targets (which start with a stressed unit) and *vice versa*. This is clearly not what we found, since the same pattern characterized both penultimate- and antepenultimate-stress words, and this is an indication that the segmental prime exerts its effect at an abstract phonological level.

Penultimate- and antepenultimate-stress targets exhibited the same pattern also when we manipulated stress priming. For both types of targets, reading times in Experiment 2 were slower in the incongruent than in the congruent (and the control) condition. Previous studies on word reading with visible primes have shown that the metrical structure of a word may be primed independently from its segmental content, and that such a priming occurs for both penultimate- and antepenultimate-stress targets. The results were taken as evidence that an abstract representation of the words’ metrical structure is available during reading, and can intervene during the stage of word phonological encoding (cf. Colombo and Zevin, 2009; Sulpizio et al., 2012b; Sulpizio and Job, 2013). The results of Experiment 2 allow us to better qualify those findings, by showing that: (a) the stress priming effect may be interpreted as inhibitory on the bases of the present experiment that included a control condition; (b) the effect is automatic, i.e., it is not driven by strategic mechanisms, since it emerges with masked primes as well.

We posit that the prime-target stress interference arises within the phonological output buffer of the reading system, and postulate a mechanism that activates stress information and specifies the position the stress takes in the word – for Italian words the three possibilities being either the antepenultimate, the penultimate, or the final syllable. During word reading, in the phonological output buffer, information about stress position coming from lexical and sub-lexical processing is collected and, as soon as activation for a stress pattern is reached, the system specifies the stressed syllable among those available. As for the time dynamics of the stress mismatch interference, when the stress pattern activated by the prime differs from the stress pattern required by the target there is a delay in specifying the position of stress within the available segmental sequence (i.e., the segment-to-frame association), since the currently available, incorrect stress pattern must be disengaged and the correct one must be activated. This proposal is similar to that put forward by Perry et al. (2010), who implemented a detailed system for stress assignment in their CDP++ model of bi-syllabic reading that we will further discuss below (see also Perry et al., 2014).

The finding of a pure metrical priming stands in contrast with the results reported by Roelofs and Meyer (1998) for speech production. Using a form-priming paradigm with Dutch words, unlike the present experiment, Roelofs and Meyer (1998) did not find a pure stress priming effect, and argued that the absence of such effect follows from the fact that metrical and segmental spell-out run in parallel and take the same amount of time (Roelofs and Meyer, 1998). Methodological differences between the two studies may account for the pattern. In the implicit form-priming paradigm, participants are required to learn cue-target word pairs, and to produce the target word upon presentation of a cue word. This differs from the present research that investigated reading aloud by adopting a masked priming procedure, in which participants have to read stimuli aloud within the frame activated by the prime information. Thus, the discrepancies in the results may reflect the processes involved in performing the tasks. In particular, in Roelofs and Meyer’s (1998) study episodic memory is heavily involved.

The joint manipulation of segmental and suprasegmental information of Experiments 3 and 4 shows an asymmetric pattern between antepenultimate-stress words, on the one hand, and penultimate-stress and final-stress words, on the other, and this allows us to better articulate the operations carried out by the phonological output buffer of the reading system. Moreover, such findings allow us to rule out the possibility that the dissociation is due to the asymmetric distribution of the stress patterns in Italian as final-stress words are quite infrequent, thus patterning with antepenultimate-stress words on this dimension, but showing no difference between the congruent and the incongruent condition, just like penultimate-stress words. As a consequence, the asymmetry among different stress patterns has to be ascribed to the temporal dynamics of the operations the reading system carries out for the stimulus.

The pattern we found may be accounted for by the UIASAP proposal, which makes three assumptions about the functioning of the phonological output buffer: (a) for words with unpredictable stress, the phonological encoding requires specifying which of the syllables receives stress among the available segmental material (see Roelofs, 2015) (b) the phonological-to-phonetic mapping takes place through a rightward incremental process (Levelt et al., 1999; for the same proposal in reading: Kinoshita, 2000; cf. Carreiras et al., 2005), with the minimal planning unit that goes from the word beginning up to (at least) the stressed syllable; (c) the reading system starts the planning of articulation as soon as the relevant information for the to-be-planned unit is active. Taken together these assumptions allow for the different temporal dynamics in the phonological output buffer for stress-related word classes. For antepenultimate-stress words, the first syllable comprises the activation of both its phonemes and the stress pattern, the latter being specified in the segmental sequence by the stress system; instead, for penultimate-stress words the first syllable comprises the activation of only its phonemes and it is the second syllable that requires the activation of both its phonemes and the stress pattern; for the final stress, the syllable requiring stress activation will be the last one. If information about the stressed syllable is needed to start articulation, for antepenultimate-stress targets articulation may start as soon as the first syllable is encoded since both segmental and suprasegmental information is available, while for penultimate- and final-stress targets articulation may only begin when information about the second or the third syllable, respectively, becomes available. Therefore, the inconsistent stress prime would affect differently the three types of words: for antepenultimate-stress words the interference would be stronger as it would directly impact on the to-be-articulated unit while for penultimate- and final-stress words there would be time to mitigate the impact of the incongruent stress prime for articulation cannot start until the information about stress become available on the second or third syllable, respectively.

The UIASAP proposal would predict an advantage for stimuli bearing earlier stress compared with those stimuli bearing later stress. The empirical evidence on this issue is scanty, with studies generally reporting contrasting evidence on reading times of penultimate- and antepenultimate-stress words. Recently, in a pseudoword reading study, an advantage for antepenultimate- over penulimate-stress targets has been reported by Sulpizio et al. (2015), who found that participants read pseudowords faster when they assigned antepenultimate than penultimate stress. Sulpizio et al. (2015) proposed that stress computation affects naming speed at the stage of articulatory planning, as readers may buffer a partial articulatory representation of stimuli that proceeds from the first syllable up to the stressed syllable (for a similar perspective, see also Sternberg et al., 1988; Laudanna et al., 1989; Sulpizio and Colombo, 2013; Sulpizio et al., 2013). For words, a similar result has been reported by Burani and Arduino (2004, Experiment 2), who showed that low-frequency antepenultimate stress words were read faster than low-frequency penultimate stress words. Note, however, that an opposite pattern has also been reported (Colombo, 1992). Finally, Burani et al. (2014) reported no difference between words with penultimate and antepenultimate stress. Thus, an antepenultimate-stress advantage appears to be elusive and difficult to detect.

Although a difference between antepenultimate- and penultimate-stress targets might have been expected in our study, we believe that there are at least two reasons to account for its absence. First, in our study the system has to process a prime-target event instead of a simple target event, with the consequence that the operations involved in the former are partly different from those involved in the latter (Kinoshita and Norris, 2012). Specifically, the “disengagement” from the prime might (globally) interfere more with words that can be articulated faster than with words that requires more time to be articulated. Therefore, the prime-target computation may obscure or take away the possible advantage of antepenultimate words. Second, and more generally, the process of lexicalization (reading aloud) is affected by several concurrent factors that weight differently for the different stress patterns. Thus, the presumed advantage for antepenultimate stress may be diminished or eliminated by the distributional properties of stress (80% of polysyllable words bear penultimate stress) and/or explicit stress marks (available only for final stress words).

A computational account for our results may be offered by the CDP++.Italian model of polysyllable reading (Perry et al., 2014), which is the Italian version of the CDP++ (Perry et al., 2010). The model implements a detailed phonological output buffer composed of two distinct mechanisms for segmental and suprasegmental computation, i.e., *Phonological Output Nodes* (henceforth PONs) and *Stress Output Nodes* (henceforth SONs). Both PONs and SONs receive activation from the lexical and the sub-lexical route in parallel and combine the two sources of information through competitive interactions. The activation within the SONs is also regulated by a lateral inhibition parameter and the activation of a stress node inhibits the other node. The CDP++ model could deal quite easily with the pure segmental and suprasegmental priming effects we reported in Experiments 1 and 2. The English version of the model has already successfully simulated the MOPE (Perry et al., 2010; see also Perry et al., 2007); the CDP++.Italian has simulated the stress priming effect reported by Sulpizio et al. (2012b) for visible priming experiments and for this reason we assume the model should be able to simulate the prime-target stress interference we reported in Experiment 2. However, *prima facie*, the CDP++.Italian does not seem equipped to account for the asymmetric pattern arising from the joint manipulation of stress and phonemes, mainly because the current implementation does not specify how the PONs and the SONs communicate with each other and this underspecifies how phonemes and stress information are assembled together. On this issue, one possibility is that the segment-to-frame association would work rightward incrementally and the phonological-to-phonetic mapping may start as soon as the relevant usable information becomes available. Therefore, while the activation within the PONs and the SONs may proceed in parallel and quite independently, the phonological-to-phonetic interface would require all the relevant (segmental and suprasegmental) information for the to-be-articulated unit to be available for the system.

On a related issue, as it stands now, the phonological output buffer of the CDP++ binds the start of articulation only to the activation of the correct stress pattern of the stimulus: according to the *stress naming criterion* parameter, independently of how easy and/or fast the word’s phonemes are being processed, reading aloud can start only after the stress has been assigned. However, the results we obtained in Experiments 3 and 4 suggest that the timing of word articulation is affected not only by stress activation, but also by phonemic activation and by the interaction between the two types of information.

## Conclusion

Our findings shed new light on the stages of phonological and phonetic encoding in word reading. We have shown that readers may compute stress apart from phonemes and that the two types of information may be independently primed as we obtained both pure segmental priming and pure suprasegmental priming in our first two experiments. The data are consistent with previous findings reported in literature (e.g., Forster and Davis, 1991; Colombo and Zevin, 2009; Dimitropoulou et al., 2010; Sulpizio et al., 2012b, for the suprasegmental priming) and provide further support for the assumption that the latest stages of reading aloud include a process of segment-to-frame association that drives the stimulus phonetic encoding (see also the speech planning account: Kinoshita, 2000; Malouf and Kinoshita, 2007). Moreover, we propose that the phonological buffer of the reading system acts as the locus of the phonological-to-phonetics interface, that is the locus where the abstract phonological word is converted into its phonetic representation as soon as the relevant information for the to-be-planned unit becomes available.

## Conflict of Interest Statement

The authors declare that the research was conducted in the absence of any commercial or financial relationships that could be construed as a potential conflict of interest.

## Footnotes

1 Capital letters indicate the stressed syllable.

2 The unique Italian rule for stress assignment requires to assign penultimate stress to those words that have a heavy penultimate syllable (e.g., biSO**N**te ‘bison’). The rule shows also some exceptions (e.g., MANdo**r**la ‘almond,’ LEpa**n**to).

3 The remaining 2% of three-syllable words bear stress on the final syllable, and in this case stress it is graphically marked (e.g., *colibrì*).

4 The analyses were also run with the block order as fixed factor. As the pattern of results was the same, we decided not to report such analyses. The analyses were also run with the block order as fixed factor. As the pattern of results was the same, we decided not to report such analyses. The same was done for all experiments.

5 The activation of phonetic rather than phonological units would be compatible with our pattern only by assuming that stressed and unstressed syllables are each activated and roughly at the same time, with the additional assumption that the stress-inconsistent syllable does not interfere. This alternative cannot be totally ruled out, but it seems unlikely.
