# Supplementary material for: The segment-to-frame association in word reading: early effects of the interaction between segmental and suprasegmental information
Source: Front Psychol. 2015 Oct 20;6:1612. doi: 10.3389/fpsyg.2015.01612 (PMC4612140; doi:10.3389/fpsyg.2015.01612)
Supplement: Supplementary file 1 [file Data_Sheet_1.DOCX]

**Appendix**

Prime and Target Words used in Experiment 2 and 3*.

|  |  | *Experiment 2* | | *Experiment 3* | |
| --- | --- | --- | --- | --- | --- |
| *Targets* | *Stress Type* | *Congruent prime* | *Incongruent Prime* | *Congruent prime* | *Incongruent Prime* |
| bolide (racing car) | a. | sinodo  (holy sinod) | sapone  (soap) | botola  (trapdoor) | bovaro  (cowherd) |
| calamo  (quill) | a. | tacito  (tacit) | sedile  (seat) | carica  (charge) | cabina  (cabin) |
| calice  (stem glass) | a. | tubero  (tuber) | sirena  (siren) | canone  (rule) | canale  (channel) |
| civico  (civic) | a. | vigile  (policeman) | tafano  (gadfly) | cinema  (cinema) | cinese  (Chinese) |
| colica  (colic) | a. | botola  (trapdoor) | tutore  (tutor) | cofano  (hood) | conato  (retch) |
| dedica  (inscription) | a. | cofano  (hood) | cabina  (cabin) | debole  (weak) | deluso  (disappointed) |
| docile  (tame) | a. | carica  (charge) | bovaro  (cowherd) | domino  (dominoes) | dogana  (customs) |
| fecola  (potato starch) | a. | cinema  (cinema) | canale  (channel) | fegato  (liver) | ferita  (wound) |
| fonico  (phonic) | a. | lecito  (right) | vinile  (vinyl) | fodera  (lining) | folata  (rush) |
| genero  (son-in-law) | a. | debole  (weak) | cinese  (Chinese) | gemito  (moan) | gelato  (ice-cream) |
| lacero  (ripped) | a. | domino  (dominoes) | conato  (hood) | lapide  (gravestone) | latino  (latin) |
| lemure  (lemur) | a. | mogano  (mahogany) | deluso  (hood) | lecito  (right) | letale  (lethal) |
| mitilo  (mussel) | a. | fodera  (lining) | dogana  (customs) | misero  (miserable) | minuto  (minute) |
| monito  (warning) | a. | gemito  (moan) | ferita  (wound) | mogano  (mahogany) | moroso  (defaulting) |
| patina  (coat) | a. | lapide  (gravestone) | folata  (rush) | papero  (duck) | pavone  (peacock) |
| remora  (hesitation) | a. | canone  (rule) | gelato  (ice-cream) | reduce  (survivor) | regata  (regatta) |
| rivolo  (rivulet) | a. | misero  (miserable) | latino  (latin) | rigido  (stiff) | ritiro  (retirement) |
| ruvido  (coarse) | a. | fegato  (liver) | letale  (lethal) | rucola  (arugula) | rugoso  (wrinkled) |
| salice  (willow) | a. | papero  (duck) | minuto  (minute) | saturo  (overfilled) | sapone  (soap) |
| setola  (bristles) | a. | reduce  (survivor) | moroso  (defaulting) | senape  (mustard) | sedile  (seat) |
| sigaro  (cigar) | a. | rigido  (stiff) | Pavone  (peacock) | sinodo  (holy sinod) | sirena  (siren) |
| tanica  (tank) | a. | rucola  (arugula) | regata  (regatta) | tacito  (tacit) | tafano  (gadfly) |
| tumulo  (mound) | a. | saturo  (overfilled) | ritiro  (retirement) | tubero  (tuber) | tutore  (tutor) |
| vipera  (viper) | a. | senape  (mustard) | rugoso  (wrinkled) | vigile  (policeman) | vinile  (vinyl) |
|  |  |  |  |  |  |
| bobina | p. | sapone  (soap) | sinodo  (holy sinod) | bovaro  (cowherd) | botola  (trapdoor) |
| calura | p. | sedile  (seat) | tacito  (tacit) | cabina  (cabin) | carica  (charge) |
| carota | p. | sirena  (siren) | tubero  (tuber) | canale  (channel) | canone  (rule) |
| cicuta | p. | tafano  (gadfly) | vigile  (policeman) | cinese  (Chinese) | cinema  (cinema) |
| colera | p. | tutore  (tutor) | botola  (trapdoor) | conato  (hood) | cofano  (hood) |
| deriva | p. | cabina  (cabin) | cofano  (hood) | deluso  (hood) | debole  (weak) |
| doloso | p. | bovaro  (cowherd) | carica  (charge) | dogana  (customs) | domino  (dominoes) |
| fenice | p. | canale | cinema | ferita | fegato |
| focoso | p. | vinile  (vinyl) | lecito  (right) | folata  (rush) | fodera  (lining) |
| genoma | p. | cinese  (Chinese) | debole  (weak) | gelato  (ice-cream) | gemito  (moan) |
| lacuna | p. | conato  (hood) | domino  (dominoes) | latino  (latin) | lapide  (gravestone) |
| legume | p. | deluso  (hood) | mogano  (mahogany) | letale  (lethal) | lecito  (right) |
| mimosa | p. | dogana  (customs) | fodera  (lining) | minuto  (minute) | misero  (miserable) |
| monile | p. | ferita  (wound) | gemito  (moan) | moroso  (defaulting) | mogano  (mahogany) |
| patata | p. | folata  (rush) | lapide  (gravestone) | pavone  (peacock) | papero  (duck) |
| remoto | p. | gelato  (ice-cream) | canone  (rule) | regata  (regatta) | reduce  (survivor) |
| ricamo | p. | latino  (latin) | misero  (miserable) | ritiro  (retirement) | rigido  (stiff) |
| rubino | p. | letale  (lethal) | fegato  (liver) | rugoso  (wrinkled) | rucola  (arugula) |
| sagace | p. | minuto  (minute) | papero  (duck) | sapone  (soap) | saturo  (overfilled) |
| semita | p. | moroso  (defaulting) | reduce  (survivor) | sedile  (seat) | senape  (mustard) |
| siluro | p. | pavone  (peacock) | rigido  (stiff) | sirena  (siren) | sinodo  (holy sinod) |
| tapino | p. | regata  (regatta) | rucola  (arugula) | tafano  (gadfly) | tacito  (tacit) |
| tucano | p. | ritiro  (retirement) | saturo  (overfilled) | tutore  (tutor) | tubero  (tuber) |
| virata | p. | rugoso  (wrinkled) | senape  (mustard) | vinile  (vinyl) | vigile  (policeman) |

*Note*: The column *Stress type* refers to the stress of the targets; a. = antepenultimate stress; p. = penultimate stress.

*Targets were also targets in Experiment 1.

Prime and Target Words used in Experiment 4

| *Targets* | *Stress Type* | *Congruent prime* | *Incongruent Prime* |
| --- | --- | --- | --- |
| cabala  (cabala) | a. | cavolo  (cabbage) | cafone  (boor) |
| carica  (charge) | a. | canapa  (hemp) | canoro  (singing) |
| conico  (conic) | a. | cotica  (pork rind) | colite  (colitis) |
| dedalo  (maze) | a. | delega  (mandate) | decano  (dean) |
| genesi  (genesis) | a. | genero  (son-in-law) | feloso  (jealous) |
| gomito  (elbow) | a. | gotico  (gothic) | Goloso  (greedy) |
| milite  (soldier) | a. | minimo  (minimal) | minore  (minor) |
| nobile  (noble) | a. | nomade  (nomad) | nodino  (knot) |
| pagina  (page) | a. | pavido  (fearfull) | papiro  (papyrus) |
| resina  (resin) | a. | regola  (rule) | regime  (regime) |
| revoca  (revocation) | a. | recita  (recital) | regalo  (present) |
| sagoma  (outline) | a. | sadico  (sadist) | savana  (savannah) |
| sesamo  (sesame) | a. | secolo  (century) | senato  (senate) |
| vivido  (vivid) | a. | vicolo  (alley) | vivace  (lively) |
|  |  |  |  |
| civile  (civil) | p. | dicala  (cicada) | cinico  (cynical) |
| cotone  (cotton) | p. | codino  (pony tail) | comico  (funny) |
| fanale  (light) | p. | favore  (favor) | favola  (fairy tale) |
| farina  (flower) | p. | famoso  (famous) | Facile  (easy) |
| felino  (feline) | p. | feroce  (ferocious) | federa  (pillowcase) |
| marina  (navy) | p. | madama  (madame) | manico  (handle) |
| misura  (measure) | p. | mirino  (gunsight) | mitico  (mythical) |
| motore  (engine) | p. | moneta  (coin) | nobile  (aristocrat) |
| rapina  (robbery) | p. | radura  (clearing) | rafano  (horseradish) |
| rivale  (enemy) | p. | rigato  (lined) | ripido  (steep) |
| salame  (salami) | p. | safari  (safari) | satiro  (satyr) |
| sifone  (syphon) | p. | sicuro  (safe) | simile  (similar) |
| tutela  (protection) | p. | tumore  (tumor) | tubero  (tuber) |
| vapore  (gas) | p. | varano  (monitor lizard) | valico  (passage) |
|  |  |  |  |
| caribù  (caribou) | f. | calerà  (she/he will lower) | capivo  (I understood) |
| carità  (generosity) | f. | calunniò  (she/he defamed) | calibra  (she/he bores) |
| casinò  (casino) | f. | cavalcò  (she/he rode) | camuffa  (she/he hide) |
| cavità  (hole) | f. | carezzò  (she/he caressed) | candiva  (she/he candied) |
| cecità  (blindness) | f. | celerò  (I will conceal) | cedono  (they collapse) |
| colibrì  (hummingbird) | f. | coverà  (it will brood) | coprono  (they cover) |
| facoltà  (faculty) | f. | faticò  (she/he toiled) | fasciavo  (I bound) |
| gigolò  (gigolò) | f. | Girerà  (she/he will turn) | giocavo  (I played) |
| levità  (lightness) | f. | lenirò  (I will relieve) | legano  (they fasten) |
| libertà  (freedom) | f. | limerò  (I will smooth) | limito  (I limit) |
| lunedì  (Monday) | f. | lucidò  (she/he buffed) | lustrano  (they shine) |
| manitù  (manitou) | f. | macinò  (she/he ground) | macera  (she/he macerates) |
| novità  (news) | f. | Nominò  (she/he named) | noleggia  (she/he rents) |
| nudità  (nakedness) | f. | nutrirò  (I will feed) | numera  (she/he enumerate) |
| parità  (equality) | f. | parlerò  (I will talk) | parano  (they block) |
| podestà  (podestà) | f. | postulò  (postulated) | potenzio  (I increase) |
| povertà  (poverty) | f. | poserò  (I will place) | potano  (they prune) |
| purità  (purity) | f. | punirò  (I will punish) | pulivo  (I cleaned) |
| rarità  (rarity) | f. | rapinò  (she/he stole) | radono  (they shave) |
| rococò  (rococo) | f. | rovinò  (she/he ruined) | rosica  (she/he/it nibbles) |
| sanità  (health system) | f. | salutò  (she/he said hallo) | sabota  (she/he sabotages) |
| separè  (private room) | f. | Seminò  (she/he sowed) | sedeva  (she/he seated) |
| serietà  (seriousness) | f. | separò  (she/he split) | sedavo  (I sedated) |
| vanità  (vanity) | f. | vagirò  (I will wail) | vagava  (she/he wandered) |
| vastità  (vastness) | f. | Valicò  (she/he crossed) | vacillo  (I wave) |
| venerdì  (Friday) | f. | veglierà  (I will watch) | veleggia  (she/he sailed) |
| verità  (truth) | f. | vegetò  (she/he vegetated) | venera  (she/he venerates) |
| voluttà  (delight) | f. | voterò  (I will vote) | vogano  (they row) |

*Note*: The column *Stress type* refers to the stress of the targets; a. = antepenultimate stress; p. = penultimate stress; f. = final stress.
